# How Estrogen, Testosterone, and Sex Differences Influence Serum Immunoglobulin Isotype Patterns in Mice and Humans

**DOI:** 10.3390/v15020482

**Published:** 2023-02-09

**Authors:** Sherri L. Surman, Bart G. Jones, Rhiannon R. Penkert, Robert E. Sealy, Tony Marion, Paul G. Thomas, Geoffrey Neale, Beisi Xu, Julia L. Hurwitz

**Affiliations:** 1 Department of Infectious Diseases, St. Jude Children’s Research Hospital, Memphis, TN 38105, USA; 2 Department of Microbiology, Immunology and Biochemistry, University of Tennessee Health Science Center, Memphis, TN 38163, USA; 3 Department of Immunology, St. Jude Children’s Research Hospital, Memphis, TN 38105, USA; 4 Hartwell Center, St. Jude Children’s Research Hospital, Memphis, TN 38105, USA; 5 Center for Applied Bioinformatics, St. Jude Children’s Research Hospital, Memphis, TN 38105, USA

**Keywords:** male, female, estrogen, testosterone, immunoglobulin isotypes, IgG2b, IgG2

## Abstract

Females often exhibit superior immune responses compared to males toward vaccines and pathogens such as influenza viruses and SARS-CoV-2. To help explain these differences, we first studied serum immunoglobulin isotype patterns in C57BL/6 male and female mice. We focused on IgG2b, an isotype that lends to virus control and that has been previously shown to be elevated in murine females compared to males. Improvements in IgG2b serum levels, and/or IgG2b ratios with other non-IgM isotypes, were observed when: (i) wildtype (WT) female mice were compared to estrogen receptor knockout mice (IgG2b, IgG2b/IgG3, IgG2b/IgG1, and IgG2b/IgA were all higher in WT mice), (ii) unmanipulated female mice were compared to ovariectomized mice (IgG2b/IgA was higher in unmanipulated animals), (iii) female mice were supplemented with estrogen in the context of an inflammatory insult (IgG2b and IgG2b/IgG3 were improved by estrogen supplementation), and (iv) male mice were supplemented with testosterone, a hormone that can convert to estrogen in vivo (IgG2b, IgG2b/IgG3, IgG2b/IgG1, and IgG2b/IgA were all improved by supplementation). We next examined data from three sets of previously described male and female human blood samples. In each case, there were higher IgG2 levels, and/or ratios of IgG2 with non-IgM isotypes, in human females compared to males. The effects of sex and sex hormones in the mouse and human studies were subtle, but frequent, suggesting that sex hormones represent only a fraction of the factors that influence isotype patterns. Examination of the gene loci suggested that upregulation of murine IgG2b or human IgG2 could be mediated by estrogen receptor binding to estrogen response elements and cytosine-adenine (CA) repeats upstream of respective Cγ genes. Given that murine IgG2b and human IgG2 lend to virus control, the isotype biases in females may be sufficient to improve outcomes following vaccination or infection. Future attention to sex hormone levels, and consequent immunoglobulin isotype patterns, in clinical trials are encouraged to support the optimization of vaccine and drug products for male and female hosts.

## 1. Introduction

### 1.1. Immunoglobulin Isotypes in Mice and Humans

In mice and humans, immunoglobulin isotypes include IgM, IgD, IgG, IgE, and IgA. IgG subtypes include IgG1, IgG2a(2c), IgG2b, and IgG3 in mice, and IgG1, IgG2, IgG3, and IgG4 in humans. The naming of isotypes and subtypes was originally based on prevalence, serological data, and physical properties [1,2,3,4,5,6,7]. The relationships between the IgG subtypes of mice and humans remain a topic of debate, because the evolutionary gene dynamics that occurred after the branching of mouse and human species are not fully understood [1,2,3,4,5,6,7,8]. Similarities between murine and human immunoglobulin isotypes include the high frequencies of human IgG2 and mouse IgG2b in carbohydrate-specific immune responses, and the partial sharing of constant region (C) gene orders within gene loci (e.g., Cγ3 is positioned upstream of Cγ1 in both species). Differences include the relatively strong binding of human IgG1, compared to mouse IgG1, to the complement subcomponent C1q [9], and a duplication in the human locus that yielded two functional Cα genes and two major regulatory regions [8].

Immunoglobulin isotypes differ in trafficking potentials and tissue residence [10,11,12], and each isotype is characterized by unique capacities for pathogen control, dictated in part by affinity/avidity for antigens, Fc receptors (including FcRn) and complement [9,13]. As examples, mouse IgGs provide protection against lung pathology and can cure influenza virus pneumonia; IgA is well known for its role in mucosal immunity, and it can prevent influenza virus transmission [10,11,14,15].

### 1.2. Differing Immune Responses and Isotype Patterns in Male and Female Hosts

Females often mount better immune responses compared to males toward vaccines and viruses including influenza virus and SARS-CoV-2 [16,17,18,19,20,21,22,23,24,25,26,27,28,29,30,31]. Estrogen plays a key role in protection [20,24,32], as was demonstrated by an improvement in vaccine responses when supplemental estrogen was administered to ovariectomized mice [32]. A partial explanation for the differences between the sexes is that female mice have higher serum immunoglobulin levels compared to males [17]. There are also significant differences in immunoglobulin isotype profiles, including higher IgG2b levels in murine females compared to males, both among total serum immunoglobulins and among virus-specific antibodies [17].

### 1.3. Estrogen Receptor (ER) Binding to the Immunoglobulin Heavy Chain Locus

Non-IgM isotype expression requires class switch recombination (CSR) to position rearranged variable (V), diversity (D), and joining (J) gene fragments near the different constant (C) region genes, either directly (e.g., CSR from Cµ to Cγ3) or sequentially (e.g., CSR from Cµ to Cγ3 and then to Cγ2b) [33,34]. Processes instrumental in CSR include: (i) RNA Pol II-mediated sterile transcription across targeted switch (S) sites and C sequences; (ii) activation-induced cytidine deaminase (AID) recruitment to positions of RNA Pol II stalling and DNA looping to juxtapose promoters, enhancers, and S regions [35,36]; (iii) DNA breaks within S sequences; and (iv) S-S ligations to delete intervening sequences and position V-D-J near non-Cµ C fragments [34,37,38].

Estrogen is a nuclear receptor ligand and may thereby influence immunoglobulin isotype profiles. Estrogen can bind and modulate the estrogen receptor (ER), which can in turn bind estrogen response elements (ERE) and CA repeats in immunoglobulin loci. CA repeats are reminiscent of heptamer/nonamer sites important for V-D-J joining, and donor/acceptor sites central to RNA splicing and intron excision [39,40,41,42]. ERE appear in human and mouse S sites and regulatory regions [17,43,44,45,46,47]. Positions of ER binding are coincident with those of other factors such as Mediator and Ikaros [35,48], known contributors to enhanceosome/switchosome activity in the locus [17]. ER and RNA Pol II exhibit coordinated binding, suggesting that ER may serve as an escort for RNA Pol II, which in turn supports sterile transcription and AID recruitment to initiate and modulate CSR [17,43,44,46,47,49,50,51,52,53,54].

### 1.4. Improving Immune Responses in Males and Females

Research policies pertinent to the development of vaccines and immunotherapies now encourage the consideration of sex as a biological variable [55]. To better define immune responses that differ between the sexes, and to help decipher pertinent mechanisms, we now describe: (i) isotype profiles in mouse models with modified levels of sex hormones, (ii) isotype profiles in males and females from three previously described clinical studies, and (iii) ERE and CA repeats upstream of mouse Cγ2b and human Cγ2 genes, sites at which estrogen and ER may influence the frequencies of CSR and C gene expression.

## 2. Materials and Methods

### 2.1. Mouse Experiments

Mice were from Jackson Laboratories (Bar Harbor, ME, USA). These included C57BL/6 male and female mice, and female estrogen receptor α knockout mice (ErαKO, strain #004744, B6,129P2-Esr1tm1ksk/). There were 5–10 mice per group per experiment unless otherwise specified. Experiments were performed in duplicate unless otherwise stated.

Mouse serum isotypes were evaluated using a Milliplex MAP Kit Mouse Immunoglobulin Isotyping Magnetic Bead Panel (Cat.#: MGAMMAG-300K) following the manufacturer’s instructions, read on a Luminex 200 instrument. Measured isotypes included IgM, IgG3, IgG1, IgG2b, IgG2a, and IgA. IgG2a values were not shown, because C57BL/6 mice express IgG2c, not IgG2a. The ratios were calculated using Excel software. When values were below or above the limit of detection (LOD), the LOD value was assigned to the sample to support graphics and statistical analyses.

#### 2.1.1. Ovariectomized Mice

Female C57BL/6 mice were ovariectomized at approximately five weeks of age at Jackson laboratories prior to shipment to St. Jude. Control female animals were unmanipulated and age-matched. Blood was collected from ovariectomized and control animals at approximately five months of age for immunoglobulin isotype analyses.

#### 2.1.2. Estrogen-Supplemented Mice

Adult female C57BL/6 mice were administered 60-day slow-release pellets of 1.7 mg 17-β estradiol (Innovative Research of America, Sarasota, FL, USA) subcutaneously (SQ) between the shoulder blades, to target release of approximately 25–30 µg supplemental estradiol per day per mouse throughout the experiment. Control mice received no supplements. Three to four weeks after the estrogen treatments were initiated, the supplemented (test) and unsupplemented (control) mice received an injection with calf thymus DNA (Invitrogen Carlsbad, CA, USA, 10 mg/mL, stored frozen at −20 °C) and peptide (NH3-KVGRRCYARLPVRASNCRKKACGHCSN-COOH, synthesized at the Hartwell Center for Biotechnology, St. Jude) in complete Freund’s adjuvant. Peptide was originally prepared and stored refrigerated as a 10 mg/mL stock in water. On the day of injection, the DNA and peptide were mixed in PBS and emulsified in an equal volume of complete Freund’s adjuvant (CFA, Thermo Scientific Rockford, IL, USA) to prepare doses of 100 µg DNA and 10 µg peptide per mouse, in a volume of 100 µL, for intraperitoneal injection. Approximately one month after the dose of DNA and peptide in CFA was administered, mice received a second dose of DNA and peptide, but in incomplete Freund’s adjuvant (Thermo Scientific Rockford, IL, USA). The prime/boost regimen was termed ‘DNA + peptide’. The DNA + peptide inoculum has been previously shown to upregulate cytokines in C57BL/6 mice, and induce autoimmune disease in some mouse strains [56,57]. Blood was collected approximately 2 weeks after the booster injection.

#### 2.1.3. Testosterone-Supplemented Mice

Adult male C57BL/6 mice received testosterone enanthate pellets (1.5 mg/pellet, 90-day release, Innovative Research of America) SQ between the shoulder blades for a targeted average release of approximately 15–35 µg testosterone/day. Control mice received no supplements. Bleeds were taken when test and control mice were approximately 4–5 months old, 2–3 months after the initiation of testosterone treatments.

### 2.2. Clinical Blood Samples and Isotyping

Samples were sera or plasma from three of our previously published studies [58,59,60], described in greater detail below. Immunoglobulin isotypes were previously described, but were not evaluated for differences between males and females. St. Jude IRB approval was received for clinical studies. Informed consent was received by adult participants and by parents/guardians of children. Assent was received from children when age-appropriate. Human isotypes were measured with the MILLIPLEX^®^ MAP human isotyping magnetic bead panel-isotyping multiplex assay (HGAMMAG-301K), as per the manufacturer’s instructions. Samples were diluted 1:16,000, and assays were performed using the manufacturer’s protocol and a Luminex 200 Multiplexing Instrument. The immunoglobulin isotype values were reported as mg/mL original serum or plasma sample. The IgG4 concentrations were generally low and were not shown. When values were below or above the LOD, the LOD value was assigned to the sample.

#### 2.2.1. Influenza Virus Study

The previously described ‘influenza virus study’ collected plasma from children and household contacts in Memphis, Tennessee (TN) after a child’s diagnosis of influenza virus infection at Le Bonheur Children’s Hospital. Children (index cases) and their household contacts (some of whom were also diagnosed with influenza virus) were monitored longitudinally [58,61]. Samples were from 19 individuals (5 males and 14 females) between 7 and 70 years of age, sampled between days 0 and 32 from the time of the index case diagnosis [58].

#### 2.2.2. Tennessee Blood Services (TBS) Study

The Tennessee Blood Services (TBS) collection was from a local blood bank in Memphis, TN that purchases human blood from adult volunteers for research purposes. The collection included 25 adult serum samples (18 males and 7 females) [58].

#### 2.2.3. FluVIT Study

The FluVIT study recruited healthy children, aged between 2 and 8 years, in Memphis, TN during the 2016–2017 influenza season [59]. Only baseline samples are described here; children later received an influenza vaccine with or without vitamin supplements. Forty baseline samples (14 males and 26 females) were evaluated.

### 2.3. Statistical Tests

Mann–Whitney tests were used to compare groups. Results for *p* values are shown in graphs when *p* ≤ 0.10. Values were not adjusted for multiple comparisons in these observational studies.

## 3. Results

### 3.1. Immunoglobulin Isotypes in ErαKO and Control Wildtype Mice

**Figure 1:**
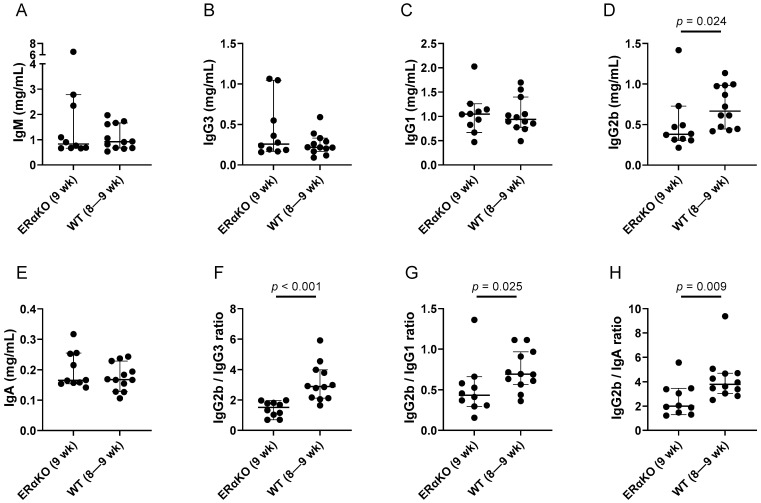
Improved ratios of IgG2b relative to other non-IgM isotypes in wildtype (WT) animals compared to ERαKO mice. Results from two sets of ERαKO and three sets of WT mice were combined for analysis. Each symbol represents a different mouse. Group medians are shown with 95% confidence intervals (CI). *p* values from Mann–Whitney tests are shown when *p* ≤ 0.1. Individual isotypes (**A**–**E**) and ratios (**F**–**H**) are shown.

To examine the effects of estrogen on serum immunoglobulin isotypes, we compared mice with a functional knockout of ER (ErαKO mice) with age-matched controls. There were two sets of test mice and three sets of control mice (2–5 mice per set), yielding ten ErαKO mice and twelve controls in total. The combined data are shown in Figure 1 and Table 1. The mice were 8–9 weeks of age. We compared the absolute isotype levels between groups, and also compared the ratios of IgG2b with other non-IgM isotypes as a measure of CSR and expression preference (IgG2b/IgG3, IgG2b/IgG1, and IgG2b/IgA). As shown, IgG2b levels were significantly higher in the control (wildtype, WT) mice compared to the ErαKO mice, and yielded significantly higher ratios of IgG2b relative to other non-IgM isotypes (IgG2b/IgG3, IgG2b/IgG1, and IgG2b/IgA).

### 3.2. Immunoglobulin Isotype Patterns in Ovariectomized Mice

**Figure 2:**
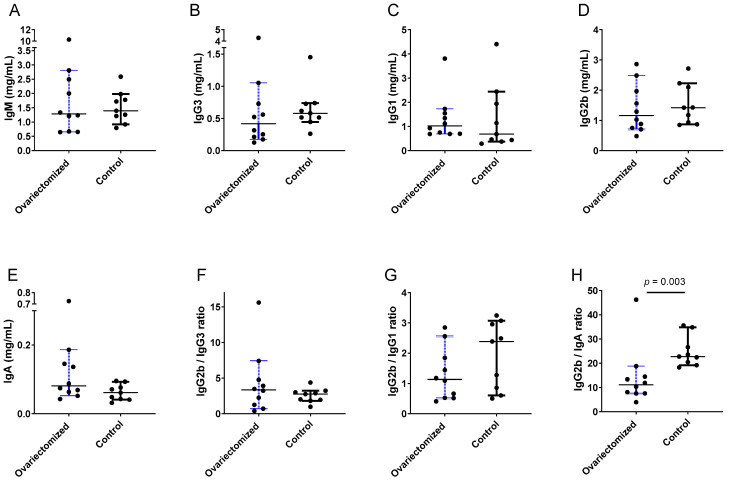
Immunoglobulin isotype patterns in ovariectomized and control mice. Each mouse is represented by a different symbol. Group medians are shown with 95% CI. *p* values from Mann–Whitney tests are shown when *p* ≤ 0.1. Individual isotypes (**A**–**E**) and ratios (**F**–**H**) are shown.

As a second strategy to examine estrogen’s influence on immunoglobulin isotypes, we compared ovariectomized C57BL/6 mice (*n* = 10) with age-matched female controls (*n* = 9). The mice were sampled at five months of age. As demonstrated by the results in Figure 2 and Table 2, the control mice exhibited marginally higher IgG2b and lower IgA compared to ovariectomized mice. This resulted in a significantly higher IgG2b/IgA ratio in the unmanipulated animals.

### 3.3. Supplemental Estrogen with an Inflammatory Insult in Female C57BL/6 Mice

**Figure 3:**
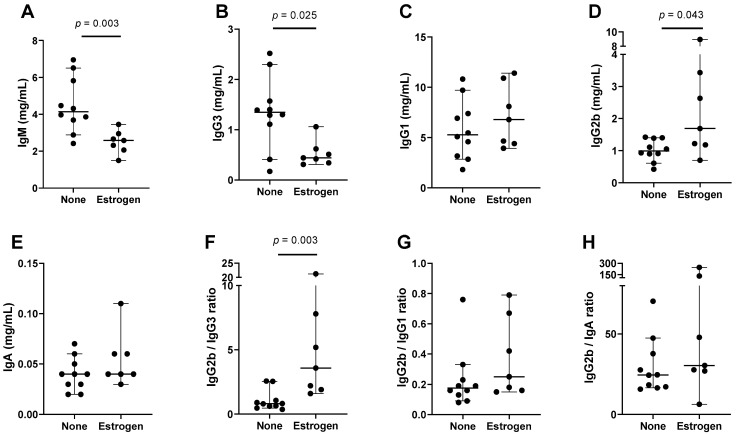
Estrogen supplementation influences isotype profiles in females in the context of an inflammatory insult. Each symbol represents a different mouse. All test and control animals received DNA + peptide. Groups only differed with respect to estrogen supplementation. Group medians are shown with 95% CI and *p* values from Mann–Whitney tests when *p* ≤ 0.10. Individual isotypes (**A**–**E**) and ratios (**F**–**H**) are shown.

As a third strategy to examine estrogen’s influence on immunoglobulin isotypes, we introduced supplemental estrogen into female mice using time-release pellets. We found that the influence of brief estrogen supplementation on serum immunoglobulin isotypes in unmanipulated, estrogen-replete females was minimal and inconsistent (not shown). However, when estrogen-supplemented and unsupplemented mice received a DNA + peptide insult, known to enhance serum cytokines [56], there were significant immunoglobulin isotype differences between the two groups. In Figure 3 and Table 3 are shown a sample experiment (ten control mice and seven estrogen-supplemented mice). As demonstrated, estrogen supplementation was associated with significant decreases in IgM and IgG3. There was significantly higher IgG2b in estrogen-supplemented mice compared to the controls. The IgG2b ratios with other non-IgM isotypes trended upward in the estrogen-supplemented mice and the change in IgG2b/IgG3 was significant.

### 3.4. Supplemental Testosterone Affects Immunoglobulin Isotype Patterns

**Figure 4:**
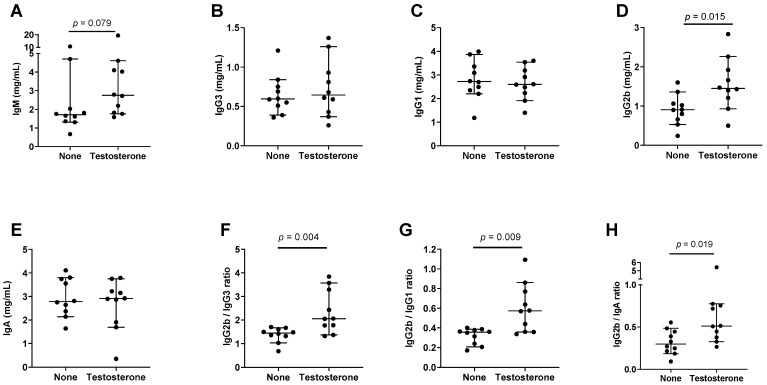
Testosterone supplements affect immunoglobulin isotype patterns in male mice. Each symbol represents a different mouse. Group medians are shown with 95% CI. *p* values from Mann–Whitney tests are shown when *p* ≤ 0.10. Individual isotypes (**A**–**E**) and ratios (**F**–**H**) are shown.

We asked if supplemental testosterone, which can convert to estrogen and augment female characteristics in males [62,63], affected immunoglobulin isotype patterns. We introduced time-release testosterone pellets into male mice and tested sera after approximately ten weeks. As shown by the experimental results in Figure 4 and Table 4 (*n* = 10 mice per group), there was a trend toward higher IgM, and a significant IgG2b increase in supplemented mice. There were also significant increases in the IgG2b/IgG3, IgG2b/IgG1, and IgG2b/IgA ratios in testosterone-supplemented male mice.

### 3.5. Human Male and Female Immunoglobulin Isotype Comparisons

**Figure 5:**
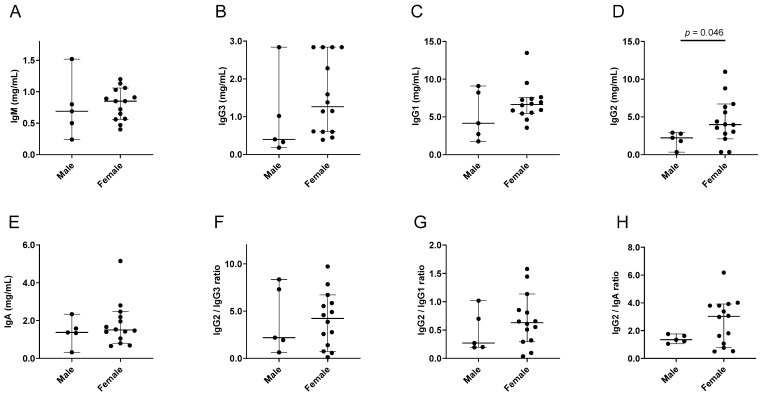
Isotype profiles in males and females in the influenza virus study. Each symbol represents a different participant. Group medians are shown with 95% CI. *p* values from Mann–Whitney tests are shown when *p* ≤ 0.10. Individual isotypes (**A**–**E**) and ratios (**F**–**H**) are shown.

We examined three sets of human data from previously published studies, all performed in Memphis, TN. Studies included (i) an ‘influenza virus study’ that recruited influenza virus-infected children and their household contacts (*n* = 19), (ii) a study of adult samples from the Tennessee Blood Services (TBS) blood bank (*n* = 25), and (iii) a FluVIT study that recruited 2–8-year-old healthy children (*n* = 40). The latter study was performed because estrogen levels are often higher in females compared to males, even in pre-pubescent children [64,65,66]. The results from the influenza virus study are shown in Figure 5 and Table 5.

As demonstrated, the females produced significantly more IgG2 compared to the males, and the ratios of IgG2 with other non-IgM isotypes showed upward trends. Because immunoglobulin isotype expression can differ between black and white populations [60], and because there were only two white participants in the influenza virus study, we examined results from the black population independently. In this case, the higher IgG2 levels were again observed in females compared to males and differences were more pronounced (*p* = 0.004 for IgG2 and 0.049 for IgG2/IgA ratios).

**Figure 6:**
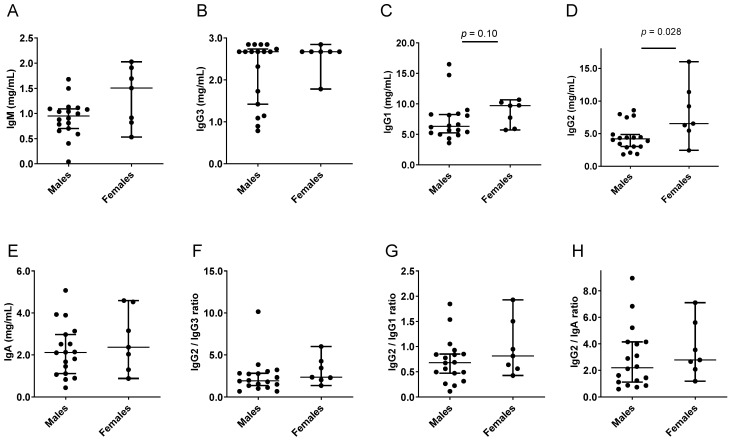
Isotype profiles in the TBS adult study. Each symbol represents a different participant. Group medians are shown with 95% CI. *p* values from Mann–Whitney tests are shown when *p* ≤ 0.10. Individual isotypes (**A**–**E**) and ratios (**F**–**H**) are shown.

Results from the TBS samples are shown in Figure 6 and Table 6. Again, IgG2 levels were significantly higher for females compared to males. There were upward trends for ratios of IgG2 compared to IgG3, IgG1, and IgA.

**Figure 7:**
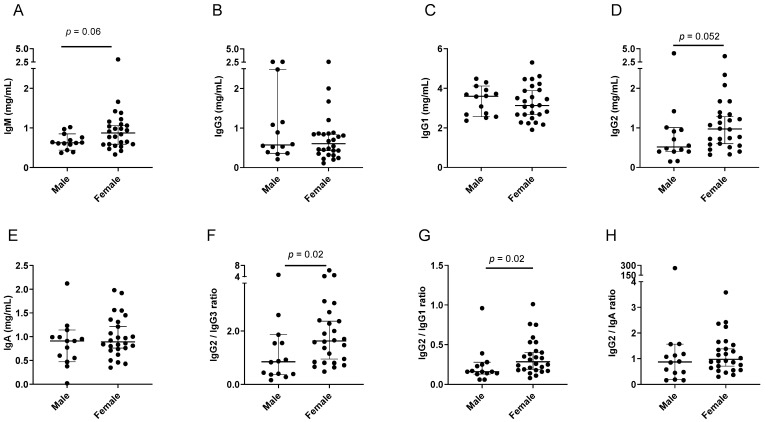
Isotype profiles in females and males within the FluVIT study. Each symbol represents a different participant. Group medians are shown with 95% CI. *p* values from Mann–Whitney tests are shown when *p* ≤ 0.10. Individual isotypes (**A**–**E**) and ratios (**F**–**H**) are shown.

Results from the FluVIT study are shown in Figure 7 and Table 7. The IgM and IgG2 differences in females compared to males approached, but did not reach, significance. The IgG2/IgG3 and IgG2/IgG1 ratios were significantly higher in females compared to males.

### 3.6. C Gene Locations in Immunoglobulin Heavy Chain Loci

**Figure 8:**
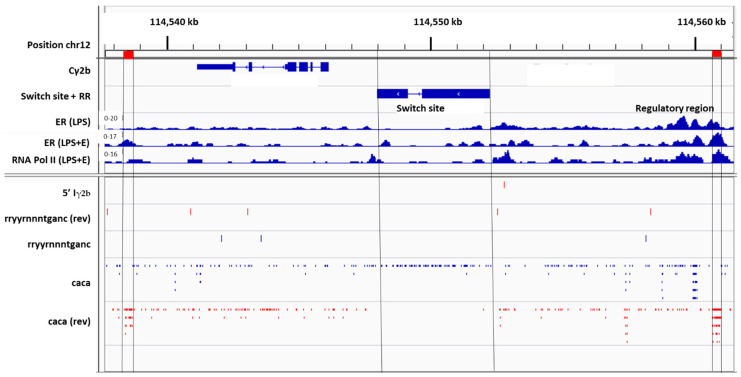
Cγ2b gene fragment and S site location in the mouse immunoglobulin heavy chain locus. The Cγ2b gene fragment is shown, oriented from right to left, using Integrative Genomics Viewer (IGV) software and the mm9 genome. ‘Find motif’ was used to identify potential EREs using the sequence rryyrnnntganc, and to identify CA repeats using the sequence, caca. ChIP experiments were with purified C57BL/6 mouse B cells stimulated for 1 day with LPS. These previously described experiments included an ER ChIP after B cell treatments with LPS (ER(LPS)), an ER ChIP after B cell treatments with LPS plus supplemental estrogen (ER(LPS + E)), and an RNA Pol II ChIP after B cell treatments with LPS plus supplemental estrogen (RNA Pol II (LPS + E)).

To understand why a bias toward IgG2b exists in female mice and a bias toward IgG2 exists in female humans, compared to males, we examined C region genes within the immunoglobulin heavy chain loci. In Figure 8 is shown the Cγ2b gene fragment oriented from right to left. Also shown is the upstream switch site, Sγ2b. Further upstream from Sγ2b is an I exon sequence (Iγ2b). The sterile transcription that precedes CSR to Cγ2b includes Iγ2b, Sγ2b, and Cγ2b sequences. A 5′ portion of the Iγ2b sequence is marked in Figure 8 (gggagagcactgggcctt).

A potential ERE (rryyrnnntganc) is positioned between 5′Iγ2b and the Sγ2b switch site. Data from previously described chromatin immunoprecipitation (ChIP) experiments are shown [17,43,46,47]. These were from LPS-stimulated, purified C57BL/6 mouse B cells that were harvested after a one-day culture. Experiments included (i) an ER ChIP after B cell stimulation with LPS, (ii) an ER ChIP after B cell stimulation with LPS + supplemental estrogen, and (iii) an RNA Pol II ChIP after B cell stimulation with LPS + supplemental estrogen. As shown in Figure 8, there were minor peaks of ER binding and RNA Pol II binding on or near 5′Iγ2b and potential ERE sequences.

Cγ2b is the first functional C gene fragment located downstream from a regulatory region termed the intermediate anchor, or γ1E [17,67]. As shown in Figure 8, this regulatory region includes numerous CA repeats, sequences that mark sites of ER binding [43,46]. The Sγ2b sequence is flanked on both sides by CA repeat hotspots.

Positions of Sγ2b and the two CA repeat hotspots are marked by vertical lines in Figure 8. CA repeats are unidirectional, but in reverse orientations when regions of Sγ2b and the CA repeat hotspots are compared (indicated by mutually exclusive blue or red dashes in the regions marked by vertical lines). Minor peaks of ER and RNA Pol II binding were identified on or near CA repeat hotspots, most notably in the upstream regulatory region (Figure 8, right). Because the regulatory region is known to influence Cγ2b expression [67], the positioning of ER and RNA Pol II in this region predicts an ER influence on DNA structure and CSR.

**Figure 9:**
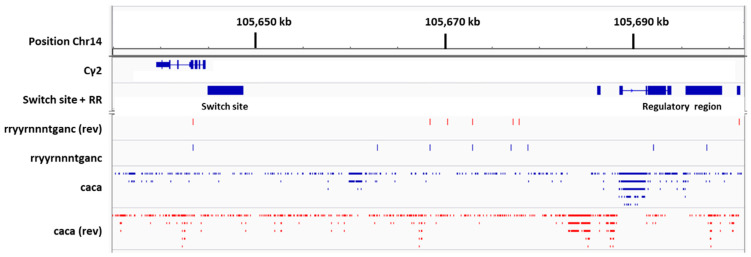
Cγ2 in the human immunoglobulin heavy chain locus. Maps were produced with the hg38 genome using IGV software. The immunoglobulin Cγ2 gene is oriented from right to left. The switch site Sγ2 is shown immediately upstream of Cγ2. Pseudogenes, not shown, exist between the regulatory region and Sγ2. ‘Find motif’ was used to identify potential ERE using the sequence rryyrnnntganc, and to identify CA repeats using the sequence, caca.

The positioning of the Cγ2 gene and its switch site in the human immunoglobulin heavy chain locus is shown in Figure 9. We did not observe an ERE immediately upstream of the Sγ2 region. The Cγ2 gene in humans is the first functional C gene segment located immediately downstream from a major regulatory region [68]. 

As for the mouse Cγ2b gene, there are prevalent CA repeats in the regulatory region upstream of Cγ2, indicating potential sites for ER binding and a consequent influence of estrogen on Cγ2 CSR and expression. The unusually high frequency of CA repeats upstream of the human Sγ2 sequence provides an environment unlike that of any other human C gene.

## 4. Discussion

### 4.1. Nuclear Receptor Ligands and Isotype Profiles

In this report, estrogen in female mice and testosterone in male mice were shown to be associated with an upregulation of IgG2b and/or IgG2b ratios with non-IgM isotypes. Biases were observed under a variety of conditions including (i) the comparison of WT and ERαKO female mice, (ii) the comparison of unmanipulated mice with ovariectomized mice, (iii) the supplementation of female mice with estrogen in the context of an inflammatory insult, and (iv) the supplementation of males with testosterone. In humans, when females were compared to males, they exhibited higher IgG2 or IgG2 ratios with non-IgM isotypes in three separate studies. The female biases in isotype usage were subtle, suggesting that sex hormones do not act in isolation. Rather, sex hormones may function in concert with a myriad of factors (e.g., vitamins [69,70,71], age, race, inflammatory stimuli) that together influence immunoglobulin isotype expression.

To help explain results, we consider the potential functions of estrogen as a nuclear receptor ligand. The binding of estrogen and ER to regulatory elements in immunoglobulin loci may directly influence CSR and gene transcription to upregulate IgG. Previous research has evaluated another nuclear receptor ligand, retinoic acid (RA), which enhances IgA production in mice and humans [58,60,72,73,74,75,76,77]. Estrogen and RA response elements overlap within S sites [44,46,69,78], providing an opportunity for receptor binding competition. In males, relatively low estrogen levels may be insufficient to compete with RA, yielding lower IgG2b levels compared to IgA [17]. This would explain the higher levels of IgG2b in males supplemented with testosterone (which can be naturally converted to estrogen, Figure 4) [62,63], and the previously described increase in IgG2b among vitamin A deficient males [17].

### 4.2. IgG Subtypes and Disease

The higher expression of IgG2b among females in mice and IgG2 in humans may help explain why females often fare better than males when vaccinated or when exposed to viruses such as influenza and SARS-CoV-2 [16,17,18,19,20,21,22,23,24,25,26,27,28,29,30,31]. In the context of COVID-19, IgG2 assists the reduction of antibody dependent enhancement (ADE) and the modulation of immune pathologies [79]. When IgG2-deficient humans were studied, patients with COVID-19 or influenza infections suffered more severe viral disease; in one study, there were more COVID-19 patients requiring mechanical ventilation, and in another study, influenza outcomes were more severe [80,81]. IgG2 has also been associated with protection from diseases caused by yellow fever virus and HIV [82,83]. In C57BL/6 mice administered a cold-adapted influenza virus vaccine, there were better virus-specific IgG2b responses in females compared to males [17]. In a separate study, when C57BL/6 mice received a purified influenza H3 hemagglutinin vaccine, the highest hemagglutination inhibition titers and neutralization titers among the IgGs were with IgG2 subtypes [84]. Perhaps a female bias toward IgG2b in mice and IgG2 in humans, although subtle, may be sufficient to improve virus control.

Unfortunately, there are negative as well as positive consequences of high levels of IgG subtypes in females, as IgGs can be associated with autoimmune disease exacerbations. Females are more likely to suffer from autoimmune diseases than males, as exemplified by the 9:1 frequency of lupus in human females compared to males [85]. Verthelyi and Ahmed found that when mice received supplemental estrogen, they produced anti-self (anti-cardiolipin) antibodies, and IgG2b was the predominant IgG subclass [86]. A separate study found that an increase in IgG1 anti-dsDNA, and/or an increase in IgG2 anti-nucleohistone antibodies, often preceded renal relapse in patients with lupus [87]. Thus, the same isotype patterns that may enhance pathogen control in females, may render females more vulnerable to autoimmune diseases.

### 4.3. Cγ2b and Cγ2 Positions in Respective Mouse and Human Immunoglobulin Gene Loci

Although IgG2b in mice and IgG2 in humans may not be direct homologues [1,2,3,4,5,6,7,8], they share functional characteristics and features within immunoglobulin loci. The Cγ2b gene in mice and the Cγ2 gene in humans are uniquely positioned downstream from regulatory regions [37,67], including ERE and concentrated CA repeats to which ER may bind. CA repeats are more concentrated upstream of the human Cγ2 gene than for any other C region gene in the human immunoglobulin heavy chain locus. Directed mutations in mice and polymorphisms in humans have revealed how isotype expression patterns depend on regulatory region sequences [35,37,38,67,88,89,90,91,92,93]. When the intermediate anchor (also termed γ1E) regulatory region was deleted in mice, IgG2b was reduced by 86.5%, a reduction that exceeded values for all other IgG subtypes [67]. ER and RNA Pol II bind ERE and CA repeats in regulatory regions where they are well positioned to influence CSR and isotype expression [17,43].

### 4.4. Limitations

A limitation of these studies was the use of a single mouse strain, C57BL/6. Mouse strains exhibit different isotype patterns and each may respond to treatments differently [17]. A second limitation was that clinical samples were only collected in one city, Memphis, TN. Variant backgrounds, environments, and diets of human hosts may influence outcomes [60,94]. As stated previously, estrogen may function interactively with other factors, including vitamins A and D, in part due to overlapping response elements in immunoglobulin loci [17,43,44,45,46,69,78,95]. A change in one factor in mice or humans is likely to alter the function of another [17]. A third limitation was that we tested only one formulation for each of the inoculations, including estrogen pellets, testosterone pellets, and DNA + peptide injections. Larger doses may have driven more dramatic changes in isotype patterns. A fourth limitation was that we did not deliberately manipulate ERE or CA sites in enhanceosomes/switchosomes in the immunoglobulin heavy chain locus to modulate the immunoglobulin isotype switch. Manipulation may be achieved by mutating ERE or CA sites in promoters, enhancers, S regions, or regulatory regions, or by designing small molecules to target response elements [96]. Perhaps deliberate manipulations will ultimately serve to improve immune responses toward pathogens and quell reactions toward self.

### 4.5. Additional Mechanisms Influence Male/Female Differences

We have focused on the potential roles of ERE and CA repeats in the immunoglobulin heavy chain locus and formulated hypotheses accordingly, but numerous additional mechanisms will impact immune differences between males and females and may help explain the experimental results. As examples, estrogen can upregulate Bcl-2 and alter the thresholds of B-cell apoptosis [97,98,99]. Testosterone is a nuclear receptor ligand and an inhibitor of inflammatory responses [100]. It inhibits BAFF, a survival factor for B cells [101,102]. Male mice lacking the androgen receptor have increased splenic B cell numbers, serum BAFF levels, and splenic BAFF mRNA. Androgens promote Tregs and suppress inflammatory cells, including dendritic cells and macrophages. These suppressive effects may worsen immune protection in some instances and quell damaging inflammatory responses and autoimmune disease exacerbations in others [22,103,104].

### 4.6. Future Prospects

Vaccines are currently produced largely as one-size-fits-all products. Some exceptions are made for children and the elderly who may receive varying vaccine doses, but host differences in nuclear hormone receptor ligands (e.g., estrogen, testosterone, vitamin A) are rarely accommodated. A better understanding of the mechanisms that distinguish male and female immune responses may eventually support the fine-tuning of vaccines and drugs for male and female recipients. Considerations for new vaccines or therapies might include the targeted stimulation of B cells with vitamins, endocrines, or endocrine-like molecules, and/or the design of synthetic molecules that target ERE or CA repeats in the immunoglobulin heavy chain locus [96].

### 4.7. Conclusions

C57BL/6 females showed a bias toward IgG2b production among non-IgM isotypes compared to males. The bias was observed when wildtype females were compared to ovariectomized or ERαKO mice. Estrogen supplements given to females in conjunction with inflammatory insults, or testosterone supplements given to males, also improved IgG2b ratios relative to other isotypes. In humans, IgG2 levels or IgG2 ratios with other non-IgM isotypes were higher in females compared to males. The subtlety of the influences emphasizes that sex hormones do not function alone, but in concert with other factors. Possibly, nuclear hormone receptors compete for binding sites within regulatory regions of the immunoglobulin heavy chain locus. The heightened IgG2b in murine females, and heightened IgG2 in human females, may help to explain why females respond better to certain viral vaccines and infections compared to males. Attention to nuclear receptor ligand levels in blood, and immunoglobulin isotype levels, may eventually support the development of better vaccines and treatments to improve immune health in male and female hosts.

## Figures and Tables

**Table 1:** Higher IgG2b in WT compared to ERαKO animals.

Isotype	n1,n2	Median ERαKO	Median WT	CI ErαKO	CI WT	*p* Value
IgG2b	10,12	0.38	0.67	0.31–0.73	0.45–0.98	**0.024**
IgG2b/IgG3	10,12	1.51	2.89	0.71–1.97	2.12–3.99	**<0.001**
IgG2b/IgG1	10,12	0.43	0.69	0.30–0.66	0.57–0.97	**0.025**
IgG2b/IgA	10,12	1.99	3.79	1.31–3.47	3.05–4.72	**0.009**

IgG2b results from Figure 1, including animal numbers (n1 for ErαKO, n2 for WT), medians, and CI values, are tabulated. *p* values are bolded when *p* < 0.05 for the ERαKO/WT comparison (Mann–Whitney test).

**Table 2:** Higher preference for IgG2b in unmanipulated (control) compared to ovariectomized female mice.

Isotype	n1,n2	Median Ovariectomized	Median Control	CI Ovariectomized	CI Control	*p* Value
IgG2b	10,9	1.16	1.42	0.71–2.49	0.87–2.23	0.60
IgG2b/IgG3	10,9	3.37	2.76	0.71–7.40	1.82–3.25	0.40
IgG2b/IgG1	10,9	1.13	2.39	0.52–2.56	0.61–3.07	0.24
IgG2b/IgA	10,9	11.15	22.74	7.54–18.77	19.16–34.79	**0.003**

IgG2b results from Figure 2, including animal numbers (n1 for ovariectomized, n2 for control), medians, and CI values, are tabulated. *p* values are bolded when *p* < 0.05 for the comparison between groups (Mann–Whitney test).

**Table 3:** Higher IgG2b in estrogen-supplemented compared to unsupplemented mice.

Isotype	n1,n2	Median No Supplement	Median Supplement	CI No Supplement	CI Supplement	*p* Value
IgG2b	10,7	0.99	1.69	0.61–1.40	0.70–8.95	**0.043**
IgG2b/IgG3	10,7	0.81	3.57	0.47–2.55	1.60–21.24	**0.003**
IgG2b/IgG1	10,7	0.18	0.25	0.09–0.33	0.15–0.79	0.22
IgG2b/IgA	10,7	24.50	30.38	16.54–47.39	6.38–245.4	0.19

IgG2b results from Figure 3, including animal numbers (n1 for unsupplemented, n2 for supplemented), medians, and CI values, are tabulated. *p* values are bolded when *p* < 0.05 for the comparison between groups (Mann–Whitney test).

**Table 4:** Higher preference for IgG2b in testosterone-supplemented compared to unsupplemented male mice.

Isotype	n1,n2	Median No Supplement	Median Supplement	CI No Supplement	CI Supplement	*p* Value
IgG2b	10,10	0.91	1.45	0.53–1.36	0.93–2.26	**0.015**
IgG2b/IgG3	10,10	1.45	2.06	1.03–1.67	1.37–3.57	**0.004**
IgG2b/IgG1	10,10	0.36	0.57	0.21–0.39	0.36–0.86	**0.009**
IgG2b/IgA	10,10	0.30	0.51	0.19–0.48	0.33–0.76	**0.019**

IgG2b results from Figure 4, including animal numbers (n1 for unsupplemented, n2 for supplemented), medians, and CI values, are tabulated. *p* values are bolded when *p* < 0.05 for the comparison between groups (Mann–Whitney test).

**Table 5:** Higher IgG2 in females compared to males in a human influenza study.

Isotype	n1,n2	Median Males	Median Females	CI Males	CI Females	*p* Value
IgG2	5,14	2.23	3.98	0.34–2.91	2.09–6.71	**0.046**
IgG2/IgG3	5,14	2.19	4.22	0.63–8.35	0.73–6.71	>0.99
IgG2/IgG1	5,14	0.27	0.63	0.19–1.02	0.29–1.14	0.44
IgG2/IgA	5,14	1.35	3.03	1.06–1.76	0.77–3.92	0.22

IgG2 results from Figure 5, including participant numbers (n1 males, n2 females), medians (mg/mL for IgG2), and CI values, are tabulated. *p* values are bolded when *p* < 0.05 for the comparison between groups (Mann–Whitney rank-based test).

**Table 6:** Higher preference for IgG2 in females compared to males in a TBS human study.

Isotype	n1,n2	Median Males	Median Females	CI Males	CI Females	*p* Value
IgG2	18,7	4.19	6.54	3.02–4.87	2.43–16.03	**0.028**
IgG2/IgG3	18,7	1.94	2.36	1.37–2.81	1.36–6.00	0.20
IgG2/IgG1	18,7	0.68	0.81	0.47–0.85	0.42–1.93	0.33
IgG2/IgA	18,7	2.20	2.78	1.13–4.15	1.19–7.10	0.39

IgG2 results from Figure 6, including participant numbers (n1 males, n2 females), medians (mg/mL for IgG2), and CI values, are tabulated. *p* values are bolded when *p* < 0.05 for the comparison between groups (Mann–Whitney rank-based test).

**Table 7:** Higher preference for IgG2 in females compared to males in the FluVIT human study.

Isotype	n1,n2	Median Males	Median Females	CI Males	CI Females	*p* Value
IgG2	14,26	0.52	0.97	0.40–1.01	0.60–1.28	0.052
IgG2/IgG3	14,26	0.85	1.63	0.37–1.86	0.95–2.37	**0.02**
IgG2/IgG1	14,26	0.16	0.29	0.13–0.28	0.19–0.40	**0.02**
IgG2/IgA	14,26	0.87	0.97	0.19–1.56	0.71–1.38	0.25

IgG2 results from Figure 7, including participant numbers (n1 males, n2 females), medians, and CI values, are tabulated. *p* values are bolded when *p* < 0.05 for the comparison between groups (Mann–Whitney rank-based test).

## Data Availability

Data will be provided upon request from authors.
